# Shear Bond Strength of Lithium Disilicate Bonded with Various Surface-Treated Titanium

**DOI:** 10.1155/2022/4406703

**Published:** 2022-04-09

**Authors:** Laongdao Amornwichitwech, Mali Palanuwech

**Affiliations:** Department of Conservative Dentistry and Prosthodontics, Faculty of Dentistry, Srinakharinwirot University, Bangkok, Thailand

## Abstract

**Purpose:**

Retention is one of the most important factors for fixed dental prostheses, especially in implant dentistry. Accordingly, the goal of this study was to evaluate the level of shear bond strength between titanium (Ti) subjected to different surface treatments and lithium disilicate glass-ceramics.

**Materials and Methods:**

In this work, 90 titanium alloy specimens were divided into six groups as follows: the control group (CT), 50 *μ*m alumina airborne-particle abrasion group (SB), silica-coated group (CJ), anodization group (AN), anodization followed by alumina 50 *μ*m airborne-particle abrasion group (ANSB), and anodization followed by silica coating group (ANCJ). Titanium specimens were bonded to lithium disilicate specimens with resin cement (Multilink N). The specimens were restored in water at 37°C for 24 h, and then, shear bond strength (SBS) tests were performed using a universal testing machine (Shimadzu, Japan). The SBS values were statistically analyzed. The failure mode of the debonded titanium was classified after viewing the samples under a stereoscope.

**Results:**

The results demonstrated that the mean SBSs of CT and AN were significantly lower than those of the other groups (*p* < 0.05). The SB group showed the highest SBS (29.47 ± 2.41 MPa); however, there was no significant difference between SB, ANSB, ANCJ, and CJ. The stereoscopic analysis demonstrated that the failure mode of AN was predominantly adhesive failure; whereas, the other groups showed cohesive and mixed failures.

**Conclusions:**

In this study, it was found that the surface treatment with 50 *μ*m alumina airborne-particle abrasion, silica coating with Cojet™ sand, anodization followed by 50 *μ*m alumina airborne-particle abrasion, and anodization followed by silica coating with Cojet™ sand improved the SBS between titanium and lithium disilicate luted with Multilink N resin cement.

## 1. Introduction

In recent years, dental implants have been the treatment of choice for replacing a single missing tooth, especially in the aesthetic zone, owing to its high clinical success [1–3]. Historically, there have been several different types of dental implant abutments used in the aesthetic zone, such as metal-colored implant abutments, tooth-colored implant abutments, and two-piece or hybrid abutments [4]. Regarding the material, titanium is commonly used as a dental implant abutment [1]. However, the metal color of the titanium can cause an unfavorable grayish appearance on gingival and lithium disilicate ceramic restorations [5–8]. Therefore, tooth-colored implant abutments such as alumina oxide and yttrium-stabilized zirconia were introduced to mitigate potential discoloration. Currently, zirconia implant abutments are widely used [9]. However, zirconia abutments can lead to wear on the internal connection of dental implant fixtures, which leads to a metal tattoo deposited into the soft tissue adjacent to the dental implant [10]. Moreover, the gap formation between a zirconia abutment and the dental implant is 3–7 times higher than that between a titanium abutment and the dental implant [11], which leads to bacterial accumulation, alveolar bone resorption [12], and abutment fracture [13]. Zirconia abutments with a small diameter also have a high fracture incidence when used in limited bone volume in the lower anterior edentulous zone. Furthermore, zirconia abutments can be easily fractured when used in limited restorative space areas such as the lower anterior edentulous ridge [14]. To address these issues, hybrid implant abutments (zirconia, lithium disilicate, or hybrid ceramic block abutments connected to a titanium core) were introduced [15, 16] to take advantage of good mechanical properties while maintaining high aesthetic properties. The use of tooth-colored and hybrid abutments is favorable in the aesthetic zone. However, two-piece abutments are more expensive than simpler one-piece titanium implant abutments. Thus, while not without drawbacks, titanium abutments appear to offer the best combination of factors [17–19]. To overcome their aesthetic problems, various coloration techniques for titanium abutments have been used. Nitride coating and anodization are common methods, but nitride has been reported to cause an allergic reaction [20].

Anodic oxidation is a titanium surface modification process that is used by many manufacturers [8]. The research on titanium anodic oxidation began in the 1950s [21]. Anodization thickens the biocompatible titanium oxide layer, which results in light reflection and color enhancement by producing a light interference pattern [19, 22]. For example, the gray hue of a metal abutment can be changed to pink or gold by varying the anodizing voltage without changing the abutment's surface biocompatibility [8, 23]. Mussano et al. claimed that pink-colored anodized titanium could improve fibroblast and epithelial cell adhesion [22]. Martinez-Rus et al. reported that the color change (Δ*E*) of a gold-colored anodized titanium abutment was comparable to that of a zirconia abutment at the soft tissue level and that tissue thickness had no effect on the Δ*E* value at the periimplant soft tissue level. As a result, in an aesthetically required area, both zirconia and gold-anodized titanium abutments could be suitable [10]. Furthermore, anodization improves the corrosion resistance [24] and may increase the surface roughness of titanium [25], which may improve the resin cement bond strength. However, little research has been conducted on the bond strength of anodized titanium and lithium disilicate glass-ceramic luting using resin cement.

Lithium disilicate glass-ceramic is widely used in restorative dentistry owing to its high aesthetic appearance [19], acceptable mechanical properties, bonding ability, and high success rate for fixed restorations [26]. This most durable glass-ceramic can be used to create a hybrid abutment with a separate crown or a hybrid abutment crown where the abutment and crown are fabricated in one piece [27]. According to Roberts et al., the fracture resistance of a complete lithium disilicate hybrid abutment crown was the highest of all the crown and abutment materials tested [28]. Since its introduction in the market in 2005, the IPS e.max Press has been recommended for fabricating inlays, onlays, and single crowns in the anterior and posterior regions. This material has a flexural strength of 350 MPa and can be used in both lost-wax and pressable techniques [29]. The same material has been developed to be used in the CAD/CAM facility. The flexural strengths of abutments fabricated using IPS e.max Press and IPS e.max CAD were reported to be similar, and the manufacturing process did not appear to affect the mechanical characteristics of the lithium disilicate ceramics. Furthermore, only the flexural strength of the CAD-processed materials was significantly influenced by translucency [30].

Another prosthetic consideration, aside from the choice of materials, is how the prosthetic crown is connected to the abutment via screw or cement-retained implant restorations. Cement-retained restorations are designed to solve the aesthetic and functional limitations of screw-retained implants, as well as the issue of implant angulation, which was less than ideal. However, excess cement may result in periimplant inflammation, especially when the margin of restoration is located deeper than 3 mm subgingivally [31]. Because of the additional components required, screw-retained restorations are frequently more expensive. Despite this, they allow for predicted retrievability [32]. At present, the techniques of “screwmentation” or “hybrid abutment crowns” combine the benefits of both techniques. The prosthesis can be cemented extraorally on the abutment using an implant analog, thus eliminating excess cement. Similar to a screw-retained crown, the final restoration is provided and torqued with retrievability [33]. One factor that can be used to quantify the clinical success rate is the bond strength between the restoration and the abutment. There are various surface treatments for enhancing the bond strength of titanium, such as nitric acid etching, alumina oxide particle abrasion, tribochemical silica coating, tin plate coating, and ND: YAG laser treatment [34]. Airborne-particle abrasion (APA) with aluminum oxide particles is widely used owing to its convenience and lower associated costs. This method increases the surface roughness and removes contamination from the titanium surface, thus strengthening the bond. Several studies [35–38] have found that sandblasting with aluminum oxide particles enhances the bond strength between titanium and ceramics. The Cojet™ system is known as a tribochemical silica coating that provides a combination of micromechanical retention and chemical bonding. First developed by 3M, this method uses APA with 30 *μ*m silica-modified aluminum oxide sand to create a reactive silica-rich outer surface. A silane coupling agent is applied, which creates cold silanization on the bonding surface. This is an effective way to enhance the bonding between metallic or ceramic surfaces and the resin cement [5, 39, 40]. Similarly, there are various surface treatments for enhancing the bond strength on glass-ceramics, such as hydrofluoric acid etching (HF) and silane coupling agents [41]. Hydrofluoric acid etching has long been used and is clinically accepted for treating silica-based ceramics [42]. The silane coupling agent provides a chemical bond between the glass-ceramic and the resin cement, allowing for the formation of a strong bond (siloxane linkage) [43]. Therefore, the combination of HF and a silane coupling agent seems to be an effective protocol to enhance the bond between the glass-ceramic and resin cement and thus reduce the risk of failure [44]. Peutzfeldt et al. compared the bond strength between human dentin and six types of dental materials using eight types of luting agents. APA titanium and HF acid-etched and silanated lithium disilicate glass-ceramics were used. In general, they found that self-etching resin cements provided higher bond strengths than other types of luting agents [45].

Nowadays, hybrid abutments and lithium disilicate crowns are prevalent [27, 46]. However, a few studies have evaluated the bond strength between lithium disilicate glass-ceramic and various surface-treated titanium alloys [47, 48]. The goal of this study was therefore to investigate the SBS between lithium disilicate and titanium alloys with various surface treatments using resin cement.

## 2. Materials and Methods

**Figure 1:**
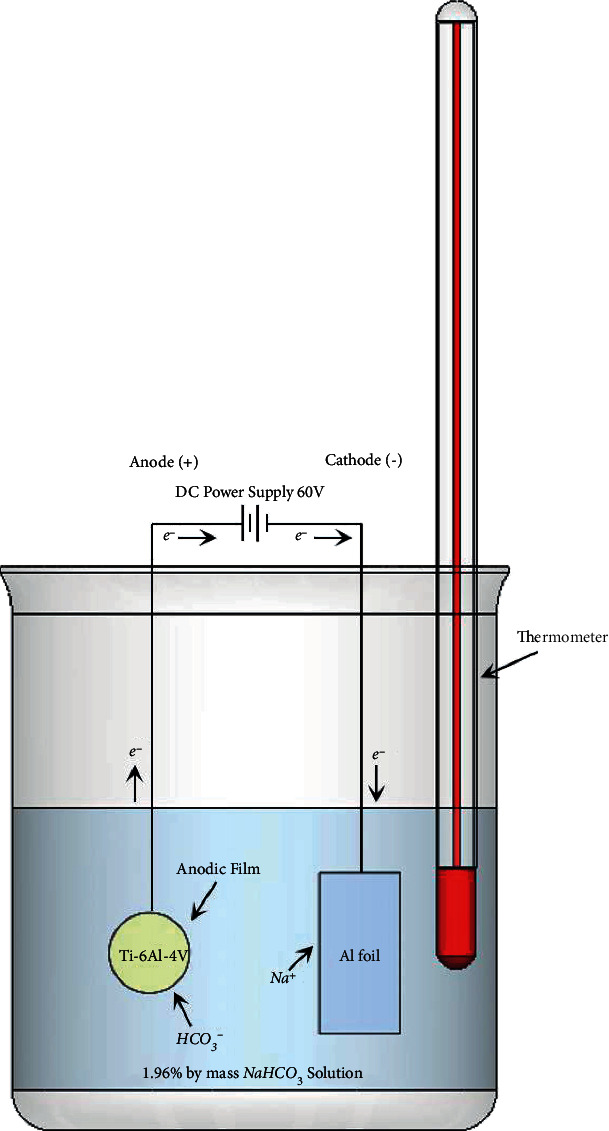
Schematic diagram of the anodizing process of titanium.

A total of 96 Ti6Al4V discs (titanium grade V; Baoji Seabird Metal Material, Shaanxi, China) with a diameter of 10.0 mm and a thickness of 3.0 mm were obtained by cutting (IsoMet 1000; Buehler, IL, USA). The titanium alloys were polished with silicon carbide abrasive papers nos. 600, 800, 1000, and 1200 (TOA; Samut Prakan, Thailand) under continuous cooling water. The polishing speed was adjusted to 100 rpm in a counter-clockwise direction, and then, the specimens were cleaned using distilled water in an ultrasonic bath (Sonorex Digitec; Bandelin, Berlin, Germany) for 10 min and dried with oil-free air. After that, forty-eight specimens were anodized using a 1.96 wt.% sodium hydrogen carbonate solution at room temperature (25°C). The Ti6Al4V discs were fixed on the anode, and the cathode was made of aluminum foil, as shown in Figure 1.

**Figure 2:**
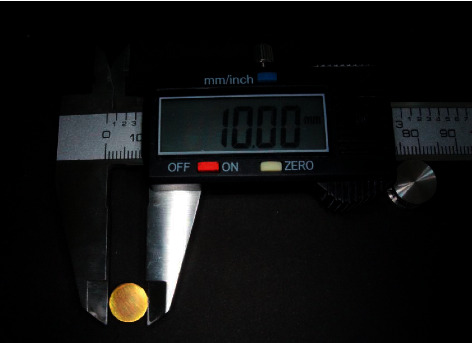
Gold-colored anodized titanium.

A fixed voltage of 60 V was supplied by a power supply machine (Switching DC Power Supply KPS1203D; Shenzhen Wanptek Electronic, Guangdong, China). The titanium specimens were submerged in the electrolyte solution for 5–10 s. At 60 V, the titanium surface presented a gold color, as shown in Figure 2. After color verification, the specimens were rinsed with distilled water.

Ninety specimens (45 bare titanium and 45 anodized titanium) were embedded in an autopolymerizing acrylic resin (Unifast™ Trad; GC America Inc., USA) inside 90 polyvinyl chloride (PVC) tubes. Six titanium alloy discs (three bare titanium and three anodized titanium) were randomly selected and kept apart for observation under a scanning electron microscope at magnifications of 500x and 1000x. All specimens were divided into six groups (15 embedded specimens and one disc specimen) according to the surface treatment methods, which were as follows:

- Control group (CT): no surface treatment on titanium surface.
- APA with 50 *μ*m aluminum oxide group (SB): APA with 50 *μ*m aluminum oxide particles (TruEtch Aluminum Oxide; Ortho Technology, West Columbia, USA) under a pressure of 2 bar at a 90° angle with 10 mm between the nozzle and the surface for 20 s using an intraoral sandblaster (MicroEtcher™ IIA; ZEST, CA, USA). The samples were then cleaned with distilled water in an ultrasonic bath for 10 min and air dried.
- Tribochemical silica coating (CJ): APA with 30 *μ*m silica-modified aluminum oxide sandblasting (Cojet™ Sand; 3M ESPE, Seefeld, Germany) under a pressure of 2 bar at 90° angle with 10 mm between the nozzle and the surface for 15 s using an intraoral sandblaster (MicroEtcher™ IIA, ZEST; CA, USA). Excess particles were gently blown off using an air drier.
- Anodization group (AN): as mentioned previously.
- Anodization followed by APA with 30 *μ*m silica-modified aluminum oxide sandblasting (ANSB)
- Anodization followed by tribochemical silica coating with 30 *μ*m silica-modified aluminum oxide sandblasting (ANCJ)

All titanium specimens were applied with a universal primer (Monobond N; Ivoclar Vivadent, Schaan, Liechtenstein). After 60 s, the remaining primer was dispersed with a strong stream of air.

Ninety lithium disilicate glass-ceramic discs (MT A1 ingot, IPS e.max Press MT ingots, A1 shade; Ivoclar Vivadent, Schaan, Liechtenstein) with a diameter of 5.0 mm and a thickness of 3.0 mm were fabricated by 3D wax printing (DentalCad 3.0 Galway; exocad GmbH Darmstadt, Germany). The wax patterns were connected with a sprue in a pressing ring, and ceramic ingots were pressed into the mold at a high temperature according to the manufacturer's instructions. The sprue was cut off with a diamond disc, and the ceramic discs were completed and polished according to the manufacturer's specifications. All lithium disilicate glass-ceramic discs were etched with 4.8% hydrofluoric acid (IPS ceramic etching gel; Ivoclar Vivadent, Schaan, Liechtenstein) for 20 s before being rinsed with running water and air dried. Monobond N was applied to the ceramic bonding surfaces for 60 s before air blowing.

The bonding area was confined to a 5 mm diameter using adhesive tape with an inner circular hole positioned on the surface of each titanium specimen. A self-curing dental luting cement with a light-curing option (Multilink N; Ivoclar Vivadent, Schaan, Liechtenstein) was applied to the bonding area using a mixing tip. The lithium disilicate specimens were loaded with a 10-N weight using a modified surveyor. The excess resin cement was removed with a microbrush before being polymerized using a light-curing unit (SmartLite® FOCUS; Dentsply Sirona, PA, USA) set to an intensity of 1000 mW/cm^2^ for 20 s/quadrant. All bonded specimens were stored in distilled water at 37°C for 24 h. The materials and resin cement used in this study are given in Table 1.

**Figure 3:**
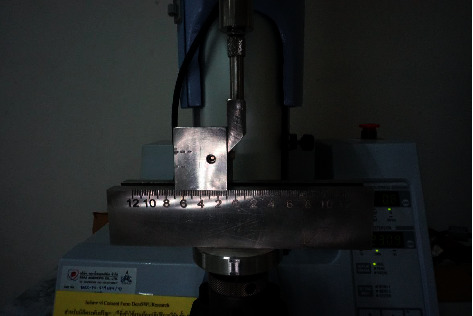
SBS test with a universal testing machine.

The bonded specimens were placed in stainless steel molds and subjected to SBS tests in a universal testing machine (Universal Testing Machine EZ-S; Shimadzu, Kyoto, Japan) with a knife-edge blade at a crosshead speed of 1 mm/min. The blade was loaded parallel between the titanium and lithium disilicate interface until rupture, as shown in Figure 3, and the SBS was recorded in newtons (N). For the SBS, *R* (MPa) was calculated from the conversion formula *R* *=* *F/A*, where *F* is the load at fracture (N) and *A* is the bonding area (mm^2^). After the bond strength test, the titanium specimens were observed under a light stereoscope (stereomicroscope; SZ61, Olympus, Tokyo, Japan) at 20x magnification. Digital images were taken for software analysis (Image *J* software Version 1.8.0; Softonic International S.A.© 1997–2021, Barcelona, Spain), and the mode of failure was allocated as adhesive, mixed, or cohesive.

Adhesive failures were characterized as having less than 25% resin cement residue on the titanium surface. Mixed failures were characterized as having more than 25% but less than 75% resin cement residue on the titanium surface. Cohesive failures were characterized as having more than 75% resin cement on the titanium surface.

One random specimen from each group was collected separately after surface treatment for scanning electron microscopy (SEM) analysis. Furthermore, each sample was photographed at magnifications of 500× and 1000× for the surface structure analysis. Finally, energy-dispersive spectroscopy (EDS) was performed for chemical element analysis.

In this investigation, we found a significant difference in the test's statistical power with 90 subjects (15 subjects per group) (G^*∗*^Power 3.1.9.4; Department of Psychology, Christian-Albrechts-University, Kiel, Germany). The Shapiro–Wilk test was used to check for a normal distribution before statistical analysis of the data using one-way ANOVA (SPSS 21.0, SPSS, Inc., Chicago, IL, USA). For multiple comparisons, Tukey's post hoc test was used with a significance level of 0.05 (*α* = 0.05).

## 3. Results

The results of the one-way ANOVA showed that the surface treatments had a significant effect on bonding (*p* < 0.05). The mean SBS values of the lithium disilicate glass-ceramic and various surface-treated titanium alloys are given in Table 2. The SB group showed the highest mean SBS value (29.47 ± 2.41 MPa), whereas the AN group showed the lowest mean SBS value (16.25 ± 2.23 MPa). The mean SBS value of the AN and CT groups was significantly lower than that of the other groups (*p* < 0.05). There were no significant differences between the SB, ANSB, ANCJ, and CJ groups (*p* > 0.05).

A light stereoscope at 20x magnification demonstrated that the failure mode of the AN group showed predominantly adhesive failure, whereas the other groups showed predominantly cohesive and mixed failures. The most prevalent among all specimens was cohesive failure (57.77%). Mixed failure accounted for 28.88% and adhesive failure accounted for 13.33%, as given in Table 3.

**Figure 4:**
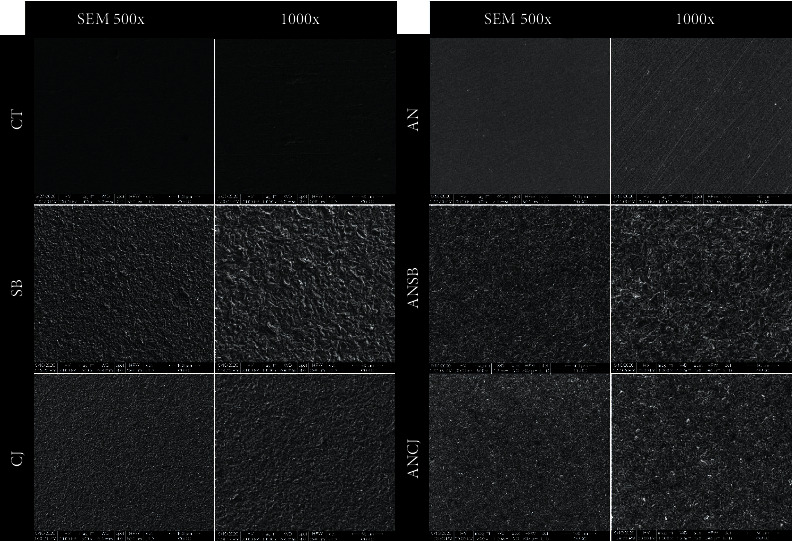
SEM images at 500x and 1000x magnification of the tested groups.

One specimen from each group was collected and analyzed using SEM (Figure 4), showing surface topography at 500× and 1000×. The elemental compositions from the EDS analysis are given in Table 4.

## 4. Discussion

The goal of this study was to evaluate the SBS between lithium disilicate bonded with various surface-treated titanium samples. The results obtained in this study prove that there is a correlation between the surface treatment of titanium and SBS. The highest value was observed in the SB group (29.26 ± 2.41 MPa), but the results were not significantly different from the CJ, ANSB, and ANCJ groups. Titanium has high chemical reactivity; thus, it is difficult to process using the lost-wax technique. At high temperatures, the reaction between titanium and other gaseous elements such as nitrogen, oxygen, and hydrogen occurs. The formation of a thick oxide layer on the Ti surface may decrease the resistance and ductility of the structure obtained [49] and interrupt the bonding capacity [50]. There have been several studies on surface treatments on titanium surfaces [34, 40, 47, 51]. The increase in the SBS of the SB group may be explained by the fact that APA with Al_2_O_3_ provided rough surfaces, increased wettability, and created a stable oxide layer [51]. It is estimated that the surface area was increased 6.5 times after sandblasting [37]. The presence of excess alumina particles decreased the bond strength between the metal and resin by decreasing the mechanical interlocking and inhibiting chemical bonding of the resin cement and the metal oxide. Therefore, alloy surfaces should be decontaminated with ultrasonic cleansing [52]. However, Al_2_O_3_ remained embedded on the titanium surfaces, and these encrusted particles establish chemical affinity between functional monomers of resin materials and themselves, increasing the resin cement's binding strength [53].

Silica-modified particles are aluminum oxide particles coated with a thin layer of silica. 3M introduced Cojet™ sand to the market, which was developed to be simple, convenient, cost-effective, and an efficient intraoral blaster for creating a reliable bond between composite and metal substructures or ceramics. Sandblasting with silica-modified alumina particles can form a tribochemical coating on the surfaces of alloys. This process is known as “cold silanization,” which enhances bond strength without thermal stress and avoids distortion of the metal framework [54]. Cojet™ sand is a silanization material that facilitates both micromechanical retention and chemical bonding mechanisms. Silane coupling agents are predominantly composed of silicon, which has the ability to chemically bond with metal ions of the superficial oxide layer of titanium [55].

There are many surface treatments that can be used to change the color of titanium surfaces, such as thermal oxidation, chemical oxidation, titanium nitride coating, and anodization. Thermal oxidation seems to have problems achieving a homogenous color and duplicability [56]. However, chemical oxidation has short-term and insufficient corrosion resistance with chemical substrates [57]. Titanium nitride coatings improve the aesthetic by changing the metal color to gold, but the layering could induce an allergic reaction [20]. Anodization is an electrolytic process that increases the thickness of the titanium oxide layer. The thickness of the anodized oxide was found to vary at different voltages, anodizing times [58], electrolyte temperatures, and types of electrolyte solution [59, 60], and the film displayed different colors depending on the light interference in the surface oxide layer [61]. TiO_2_ was found to be the major oxide on the titanium surface [62]. The titanium surface demonstrated a gold appearance at 60 V, and gold-anodized titanium provides a more favorable clinical outcome and higher patient satisfaction [63]. Therefore, anodic oxidation might be a simple, inexpensive, predictable, and ecologically favorable method [19, 24, 64]. In nature, a titanium oxide film on titanium surfaces is approximately 2–7 nm thick [62]. In this study, basic and safe laboratory techniques were considered. Therefore, a low voltage of 60 V and a sodium bicarbonate solution as the electrolytic solution were used to perform in gold-anodized titanium. Sodium bicarbonate (NaHCO_3_), or baking soda, is an alkaline salt of carbonic acid that can act as an electrolyte substitute [65], and anodizing at a voltage lower than the dielectric breaking point generates a thin (a few hundred nanometers thick), dense, amorphous oxide layer [66]. On the basis of the literature review, the thickness of the anodized film in this study was approximately 180 nm [25], with a nonporous and smoother surface than that of the nonanodized titanium, as shown in Figure 4. This is why the anodized group exhibited the lowest bond strength. Thus, we combined other surface modifications on the anodized titanium. The results of the ANSB and ANCJ groups were better than those of the AN group owing to micromechanical retention and the removal of excess oxide layers. However, after APA with either 50 *μ*m Al_2_O_3_ or 30 *μ*m modified alumina particles, the surfaces turned grayish.

There are many different arguments concerning the relationship between the particle size and bond strength. Some authors reported that larger particles had higher SBS than smaller particles [51, 67]. However, some authors found no significant difference [35, 52]. This study found no significant difference between the SB and CJ groups, even though CJ had smaller particles than SB. This can be ascribed to the chemical bonding between the silica-coated layer and the silane coupling agent in the universal primer, which created a strong and long-lasting bond [55]. However, the roughness of the metal surface plays a major role in increasing the bond strength. As seen in the SEM images under 1000x magnification (Figure 4), AN had the smoothest surface and SB had the roughest surface. This can be explained by the fact that after the anodization process according to this study's protocol, the titanium surface turned gold and smooth owing to the increased thickness of the titanium oxide layer. However, the AN group exhibited predominantly adhesive failure in accordance with the study by Akar et al [23]. Other groups suffered predominantly cohesive or mixed failures.

In addition to mechanical interlocking, chemical bonding is also a crucial factor in reinforcing the bond strength. Universal primers have become widely used in clinics and provide acceptable bond strength for all types of restoration materials, including both metals and ceramic [68]. Monobond N contains three different functional monomers: silane methacrylate (3-trimethoxysilylpropyl methacrylate), phosphoric acid methacrylate (10-MDP:10-methacryloyloxydecyl dihydrogen phosphate), and sulfide methacrylate [69]. Phosphoric derivative monomers can be well absorbed on titanium surfaces and increase the bond strength of the resin cement to the base metal. However, despite cleaning with ethyl alcohol in an ultrasonic bath, researchers found remnants of phosphorus remaining on the titanium surface [70]. The MDP monomer reacts with the metal oxide layer of titanium, including bonds to hydrogen, covalent bonds, and van der Waals forces [71]. Moreover, silane coupling agents might improve the adhesion between titanium and resin cement. Previous studies reported that silane mildly adhered to metal alloys (–Si–O–M–), but silane forms the strongest bond with silica-rich material surfaces (–O–Si–O–M), which might explain why the SB, CJ, ANSB, and ANCJ groups had similar bond strengths, even though Cojet™ sand has smaller particles than alumina oxide. Grit blasting with alumina powder enhances bonding through micromechanical interlocking, and an alumina coating layer may form on the titanium alloy surface after blasting. The amount of remaining alumina could form –Al–O–Si– linkages with the silane substrate. However, these linkages are weaker than –Si–O–Si ones [55]. Silane also increases the wettability of resin cement on the titanium surface [72]. Sulfide methacrylate has a sulfur-containing group, which is chemically bonded to noble metals [73]. Titanium was classified as base metal, so sulfide methacrylate has low affinity to titanium alloys.

In this study, there were significant differences in SBS across the four APA groups, the AN group, and the CT group. However, there was no significant difference among the SB, CJ, ANSB, and ANCJ groups. These results concur with those of other studies [74, 75], which showed a significantly higher bond strength of mechanical interlocking on the adhesion of luting cements to titanium. There was no statistically significant difference between 50 *μ*m Al_2_O_3_ APA and 30 *μ*m silica coating. The SBS of intact teeth is 13.40 MPa [76], which can represent a minimal acceptable SBS. Therefore, all groups in this study met these criteria and achieved SBS values 1.21–2.20 times higher than that of intact teeth. It may be presumed that surface treatment methods in this study resulted in stronger SBS values than that of intact teeth, and therefore, they could effectively minimize the dislodging of fixed implant prostheses in clinical situations.

The limitations of this in vitro study included the use of only one type of resin cement and primer and the use of disc-shaped specimens instead of complete restorations. This study also did not include the influence of other factors such as pH changes, temperature changes, long-term water storage, and dynamic fatigue loading, which should be investigated further to simulate actual oral cavity conditions.

## 5. Conclusion

From the limitations of this study, it can be concluded that surface treatments (SB, CJ, ANSB, and ANCJ) on titanium produced higher SBS values than the AN and CT groups because of the promotion of mechanical retention and chemical bonding between the titanium surface and resin cement. Furthermore, following AN, APA causes the surface to appear greyish, which may be seen with the bare eyes.

## Figures and Tables

**Table 1:** Materials used in this study.

Material	Composition	Manufacturer	Batch no.
Ti grade 5	Ti6Al4V	Baoji Seabird Metal Material, Shaanxi, China	B16082322
IPS e.max Press	SiO_2_, Li_2_O, K_2_O, P_2_O_5_, ZrO_2_, ZnO, and other oxides and ceramic pigments	Ivoclar Vivadent, Schaan, Liechtenstein	X43871, X47063
Aluminum oxide particles	50 *μ*m, Al_2_O_3_	TrueTech, Ortho Technology, USA	22147
Cojet™ sand	30 *μ*m, silica-modified Al_2_O_3_	3M ESPE, Seefeld, Germany	5248955
IPS ceramic etching gel	4.8% hydrofluoric acid	Ivoclar Vivadent, Schaan, Liechtenstein	Y34242
Monobond N, universal primer	Ethanol, 3-trimethoxysilylpropylmethacrylate, 10-MDP, and disulfide acrylate	Ivoclar Vivadent, Schaan, Liechtenstein	Y46574
Multilink N, resin cement	Dimethacrylate, HEMA, barium glass, yttrium trifluoride, and spheroid-mixed oxide	Ivoclar Vivadent, Schaan, Liechtenstein	Y26001

**Table 2:** Shear bond strength value between lithium disilicate and various surface-treated titanium samples (*α* = 0.05).

Test group (*n* = 15)	Mean (MPa)	SD
CT	24.28^b^	±1.60
SB	29.47^a^	±2.41
CJ	27.55^a^	±2.05
AN	16.25^c^	±2.23
ANSB	27.84^a^	±2.71
ANCJ	28.16^a^	±2.77

The same superscript letter indicates no statistically significant difference between the groups (*p* < 0.05).

**Table 3:** Modes of failure.

Group	Adhesive mode	Mixed mode	Cohesive mode
CT	0	33.33	66.67
SB	0	13.33	86.67
CJ	0	40	60
AN	73.33	20	6.67
ANSB	0	20	80
ANCJ	6.66	46.67	46.67
Sum	13.33	28.89	57.78

**Table 4:** Elemental composition (%) from EDS analysis of tested groups.

Element	CT	SB	CJ	AN	ANSB	ANCJ
Ti	85.2	32.6	38.8	68.4	44.7	38.9
Al	6.9	21.2	9.9	4.3	17.6	10.9
V	2.9	1.1	1.5	2.6	1.7	1.4
O	—	41.9	41.2	22.9	33.8	40.8
C	5	4.8	2.7	1.8	2.1	1.9
Si	—	—	6	—	—	6.1

## Data Availability

The data used to support the findings of this study are included within the article and are also be available from the corresponding author upon request.
